# Incidence and risk factors of massive subretinal hemorrhage in retinal angiomatous proliferation

**DOI:** 10.1371/journal.pone.0186272

**Published:** 2017-10-12

**Authors:** Jae Hyung Lee, Mee Yon Lee, Won Ki Lee

**Affiliations:** 1 Department of Ophthalmology and Visual Science, Seoul St. Mary’s Hospital, College of Medicine, The Catholic University of Korea, Seoul, South Korea; 2 Department of Ophthalmology and Visual Science, Uijeongbu St. Mary’s Hospital, College of Medicine, The Catholic University of Korea, Uijeongbu, South Korea; University of Utah (Salt Lake City), UNITED STATES

## Abstract

**Objective:**

To evaluate the incidence and associated risk factors of massive subretinal hemorrhage (SRH) in patients with retinal angiomatous proliferation (RAP).

**Methods:**

A total of 187 eyes of 135 treatment-naıve patients diagnosed with RAP were evaluated retrospectively. Clinical records including the time between the initial visit, last anti-vascular endothelial growth factor (VEGF) treatment, last stable examination, and the date of massive SRH were reviewed. Imaging findings including indocyanine green angiography (ICGA) and optical coherence tomography (OCT) were analyzed.

**Results:**

Massive SRH developed in 18 eyes (9.6%) a median of 20 months after the initial presentation. Kaplan-Meier survival analysis revealed that the incidence (2.8, 5.8, 13.1, and 21.0% after 1,2,5 and 10 years, respectively) continuously increased. Among 14 eyes with discernable vascular anastomosis on baseline ICGA, 13 (92.8%) showed retinal arteriole involvement. On spectral-domain OCT imaging of the last visit prior to the massive SRH, a layered lamellar tissue complex was noted under the retinal pigment epithelium in 9 of 13 eyes, which was significantly associated with massive SRH[hazard ratio(HR),5.883;P = .010]. The average time between the last stable examination/last injection and the massive SRH was 2 and 5 months, respectively. The patients were treated with anti-VEGF, gas and recombinant tissue plasminogen activator injection; however, all except one eye had visual acuity worse than 20/1000 at the final visit.

**Conclusions:**

Massive SRH can occur in RAP in the course of anti-VEGF treatment, resulting in severe vision loss. A proactive dosing regimen may be more appropriate for these RAP eyes.

## Introduction

Retinal angiomatous proliferation (RAP) is a subtype of neovascular age-related macular degeneration (nAMD) characterized by proliferation of deep retinal capillaries, with the later stages involving the progression from subretinal to choroidal neovascularization (CNV) with or without pigment epithelial detachment (PED).[1, 2] The pathogenesis of RAP is complex and the origin of the neovascular process (from the retina or from the choroid) remains controversial.[1, 3, 4] The term “type 3 neovascularization” has also been proposed for this condition to emphasize the intraretinal location of the vascular complex.[4, 5] RPE dysfunction and subsequent deposits in the subretinal space and thickening of Bruch membrane (reticular pseudodrusen) may reduce inner retinal oxygenation and upregulate angiogenic growth factor, leading to intraretinal neovascularization.[1, 6]

On angiography, RAP lesions exhibit retinal-retinal anastomosis and retinal-choroidal anastomosis, which are thought to contribute to a high blood supply to the lesions.[2] Possibly due to this high vasogenic potential, the prognosis of RAP is poor, being characterized by a rapid progression to disciform scarring without treatment.[7] In contrast, the response of RAP lesions to anti-vascular endothelial growth factor (VEGF) agents is encouraging, and studies including short to intermediate-term data have reported promising results.[8–10] However, it has been suggested that long-term visual outcomes of RAP might be limited due to the development of geographic atrophy (GA) after repeated anti-VEGF injections.[11, 12]

Another possible complication that can limit a good visual prognosis in nAMD is subretinal hemorrhage (SRH). SRH is a quite common finding in patients with nAMD and is associated with a worse visual prognosis, especially when massive subfoveal blood is present.[13, 14] Shearing stress, nutritional deprivation effects on photoreceptors by fibrin clots, and ferric toxicity from accumulated iron compounds may lead to permanent damage to the neurosensory retina and retinal pigment epithelium (RPE).[15, 16] Although massive SRH is less common among any SRH developed in nAMD,[17] it has been frequently reported in polypoidal choroidal vasculopathy (PCV) eyes as an initial manifestation, a natural course, or a complication during treatment.[18–20] However, massive SRH has never been reported as a natural course or complication during treatment of RAP, which is a distinct subtype of nAMD with characteristic vascular anastomosis and poor prognosis. Thus, we conducted a retrospective study to determine the incidence of massive SRH in RAP eyes and to evaluate the possible risk factors for its development.

## Materials and methods

We reviewed medical records and images retrospectively of consecutive treatment-naïve patients diagnosed with RAP over a period of 10 years (between June 2005 to May 2015) at Seoul St. Mary’s Hospital (known as Kangnam St. Mary’s Hospital before May 2009), The Catholic University of Korea. This study received approval from the Seoul St. Mary’s Hospital of The Catholic University of Korea Institutional Review Board and was conducted in accordance with the Declaration of Helsinki. The institutional review board waived the need for a written consent from the participants, because of the retrospective design. Patient information was anonymized and de-identified prior to analysis.

Each case was diagnosed as RAP based on characteristic features on funduscopy, fluorescein angiography (FA), indocyanine green angiography (ICGA), and optical coherence tomography (OCT), which included intraretinal hemorrhage, intraretinal vascular anastomoses, and the hyperreflective intraretinal vascular complex. Massive SRH was defined by subretinal and/or sub-RPE hemorrhages larger than a circle with a radius corresponding to the distance between the optic disc and fovea, and extending past the major vascular arcade of the retina.[21,22]

Information regarding the patient’s medical history and types and dates of treatment were collected along with clinical details of the massive SRH. The interval between the last treatment and massive SRH, and between the last stable examination and massive SRH, were analyzed. Snellen best-corrected visual acuity (BCVA), digital color fundus photographs, and OCT images were obtained at baseline and at each follow-up visit for all patients. OCT images were obtained using a Stratus (Carl Zeiss Meditec, Dublin, CA, USA) or Cirrus OCT (Carl Zeiss Meditec) before November 2011, and then with a Spectralis OCT (Heidelberg Engineering, Heidelberg, Germany). FA and ICGA (Heidelberg Retina Angiograph; Heidelberg Engineering), with a confocal scanning laser ophthalmoscope system, were performed at baseline and when required. Although Yannuzzi and associates classified the three stages of RAP, it is often difficult to differentiate whether the serous PED is associated with subretinal neovascularization (stage 2 RAP) or CNV (stage 3 RAP). Thus, in this study, RAP was classified as early stage (RAP without PED), which was equivalent to stage 1, or as advanced stage (RAP with PED) that included stages 2 and 3.

Initially, treatment-naïve RAP eyes were treated with a combination of photodynamic therapy (PDT) and bevacizumab (Avastin; Genentech, South San Francisco, CA, USA) followed by anti-VEGF injections with as-needed dosing [pro re nata (PRN)]) or anti-VEGF monotherapy from the beginning, based on PRN dosing with or without three loading injections. Both ranibizumab (Lucentis; Genentech) and bevacizumab were used, and selection between the two drugs was determined mainly by considering the national health insurance coverage and reimbursement schedules. All patients were followed regularly at 1–3-month intervals, depending on the lesion activity, and retreatment was given whenever evidence of persistent/recurrent fluid, involving or threatening the fovea, was detected on follow-up OCT. For the treatment of massive SRH, patients received anti-VEGF injections with or without gas/recombinant tissue plasminogen activator (rt-PA) injection, and all decisions were at the discretion of a single physician (WK Lee). When gas/rt-PA injection was performed, 0.1 mL (50 μg) of rt-PA and 0.2 mL of 100% C_3_F_8_ gas were intravitreally injected, followed by an anti-VEGF injection within 1 week. Patients injected with gas were instructed to remain in the prone position for as long as possible (i.e., for at least 24 h and up to 5 days). A pars plana vitrectomy was performed when a breakthrough vitreous hemorrhage occurred.

The proportion of patients who developed a massive SRH at each time point was analyzed with a Kaplan–Meier survival curve model using SPSS software (ver. 20.0; SPSS Inc, Chicago, IL). The date of initial treatment visit was designated as the index date. If a patient was lost to follow-up or did not present with a massive SRH, the latest visit was considered the last visit and it was regarded as censored data in the survival analysis. Cox regression analysis was performed to investigate the risk factors for massive SRH with clinical variables, such as age, hypertension, initial BCVA, use of PDT, and other funduscopic/OCT findings.

## Results

During the study period, in total, 187 eyes of 135 patients were diagnosed with RAP and 104 patients were females (77.0%). The mean age at onset was 75.1 ± 6.3 (range, 58–95) years and baseline logMAR BCVA was 0.51 ± 0.46 (range, 0.0–1.7). The mean follow-up period for all patients was 47.6 ± 29.0 (range, 6–133) months. During the follow-up period, 39 (20.9%) eyes received a combination of PDT and bevacizumab as the initial treatment and 148 (79.1%) eyes received anti-VEGF monotherapy. Baseline characteristics of the 187 enrolled eyes are shown in Table 1.

**Table 1:** Clinical characteristics of enrolled patients with retinal angiomatous proliferation.

No. of patients (eyes)	135 (187)
Gender (M/F) (n, %)	31 (23.0) / 104 (77.0)
Age of onset (years)	75.1 ± 6.3 (58–95)
Hypertension (n, %)	99 (52.9)
Initial BCVA (logMAR)	0.51 ± 0.46
Reticular pseudodrusen (n, %)	133 (71.1)
Use of PDT (n, %)	39 (20.9)
Fibrovascular tissue on SD OCT at final visit ^a^	96 (56.8)
Well-organized layered lamellar tissue within fibrovascular tissue on SD OCT at final visit ^a^	35 (20.7)
Mean follow-up period (months, range)	47.6 ± 29.0 (6–133)

BCVA = best-corrected visual acuity; logMAR = logarithm of the minimal angle of resolution; PDT = photodynamic therapy; SD OCT = spectral-domain OCT

^a^ analyzed in 169 eyes which underwent Spectralis OCT until final visit, and with OCT images at the last visit prior to the massive SRH in 13 eyes with massive SRH

**Fig 1:**
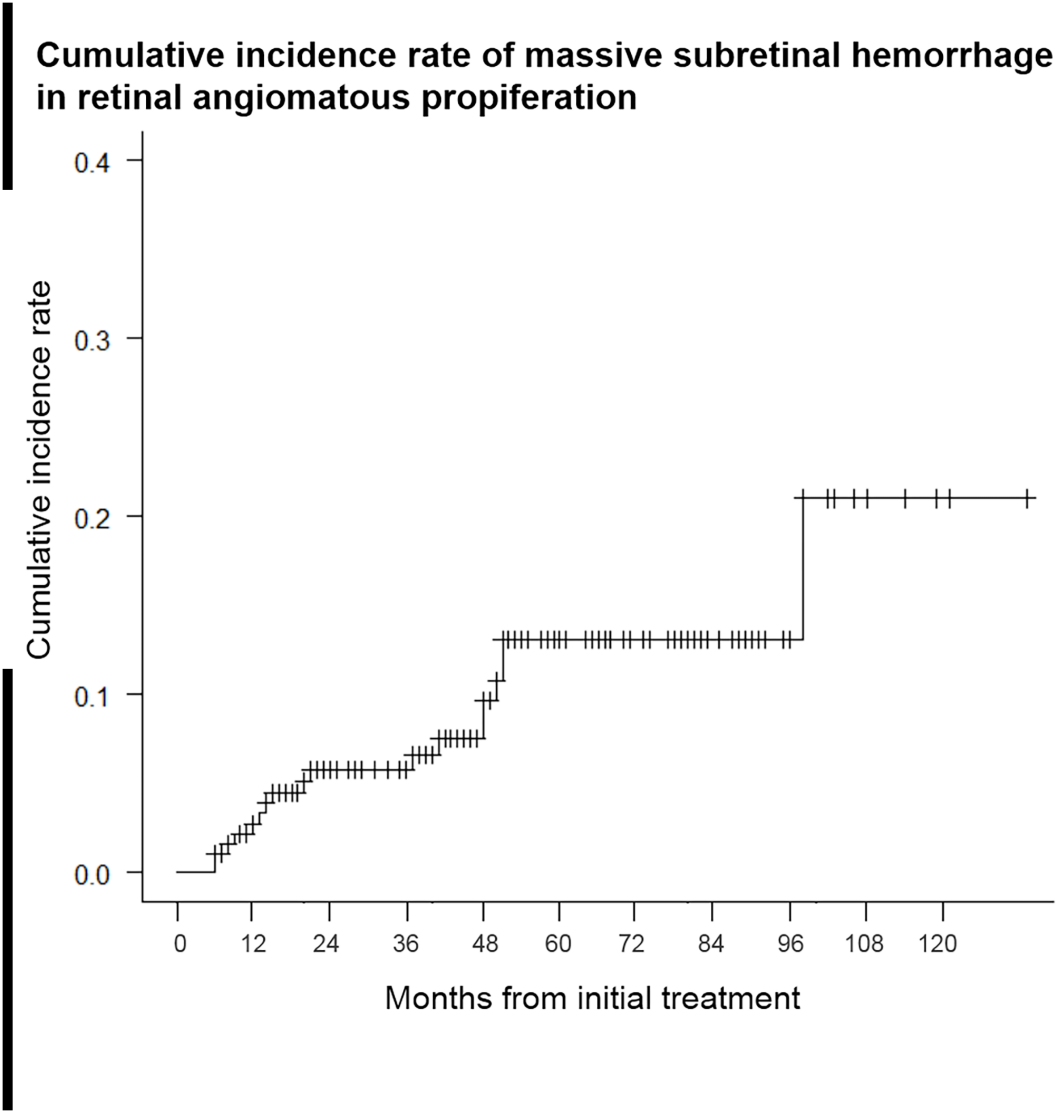
Cumulative incidence of massive subretinal hemorrhage (SRH) in retinal angiomatous proliferation (RAP) according to a Kaplan—Meier survival analysis. The horizontal axis shows the time from baseline (treatment initiation with for RAP), and the vertical axis shows the proportion of eyes that developed SRH. The incidence rates of massive SRH were 2.8%, 5.8%, 13.1%, and 21.0% of eyes at 1, 2, 5, and 10 years, respectively, after the initial treatment.

Among these eyes, RAP as a cause of massive SRH was diagnosed in 18 eyes of 17 patients; thus, the overall incidence of massive SRH was 9.6%. Massive SRH appeared after a median follow-up period of 20 (range, 6–98) months. Kaplan—Meier survival analysis revealed that 2.8% of eyes had developed massive SRH within the first year of the follow-up period. Thereafter, 5.8%, 13.1%, and 21.0% suffered massive SRH within 2, 5, and 10 years, respectively, during the follow-up period (Fig 1). Non-massive SRH (larger than 1 disc diameter, but smaller than defined as massive SRH in our method) occurred in eight eyes during the follow-up period.

The median age of the 17 patients with massive SRH was 71 (range, 64–84) years. Of them, 3 patients were males and 14 were females. Six (35.3%) patients were on aspirin therapy and no patient was using any other kind of anticoagulation therapy. The median follow-up duration was 59 (range, 24–104) months for these patients. Bilateral involvement of RAP was noted in 14 eyes during the follow-up period, and GA without neovascularization was diagnosed in the contralateral eyes of the other four eyes. Macular soft drusen within the major vascular arcade were seen in all eyes and reticular pseudodrusen in 15 (83.3%) eyes. Based on the baseline examinations, all 18 eyes were in the advanced stages (RAP with PED).

Although retinal-retinal anastomosis or retinal-choroidal anastomosis was found in all RAP lesions, their venous or arterial origin was detectable on baseline ICGA in 158 of 187 (84.5%) eyes. In the other 29 eyes, we failed to obtain enough images of the early filling sequence and/or the image quality was too poor to identify the origin of vascular anastomosis. Among 158 eyes, 75 (47.5%) showed retinal arteriole involvement on baseline ICGA and 83 (52.5%) showed involvement of retinal venules alone. The vascular component was detectable in 14 of 18 eyes with massive SRH. Among them, 13 (92.8%) eyes showed retinal arteriole involvement on baseline ICGA and one showed involvement of retinal venules only. The rate of retinal arteriole involvement was significantly higher in eyes with massive SRH (P < .001, Chi-square test).

Two patients (two eyes) received combination therapy with PDT and bevacizumab as an initial treatment, and the intervals between the last PDT and SRH were 37 and 98 months, respectively. The other 15 patients (16 eyes) were treated with anti-VEGF monotherapy. The median number of injections before the massive SRH was eight (range, 3–17), and the median time between the last injection and the massive SRH was 5 months (range, 3 days–22 months). In two eyes, massive SRH occurred within 1 month after the last anti-VEGF injection (3 days and 3 weeks, respectively). The other 16 eyes showed no sign of disease activity, such as macular hemorrhage or fluid, on the most recent examination and OCT before the occurrence of the massive SRH. The average time between the last stable examination and the massive SRH was 2 (range, 1–3) months in these 16 eyes.

**Fig 2:**
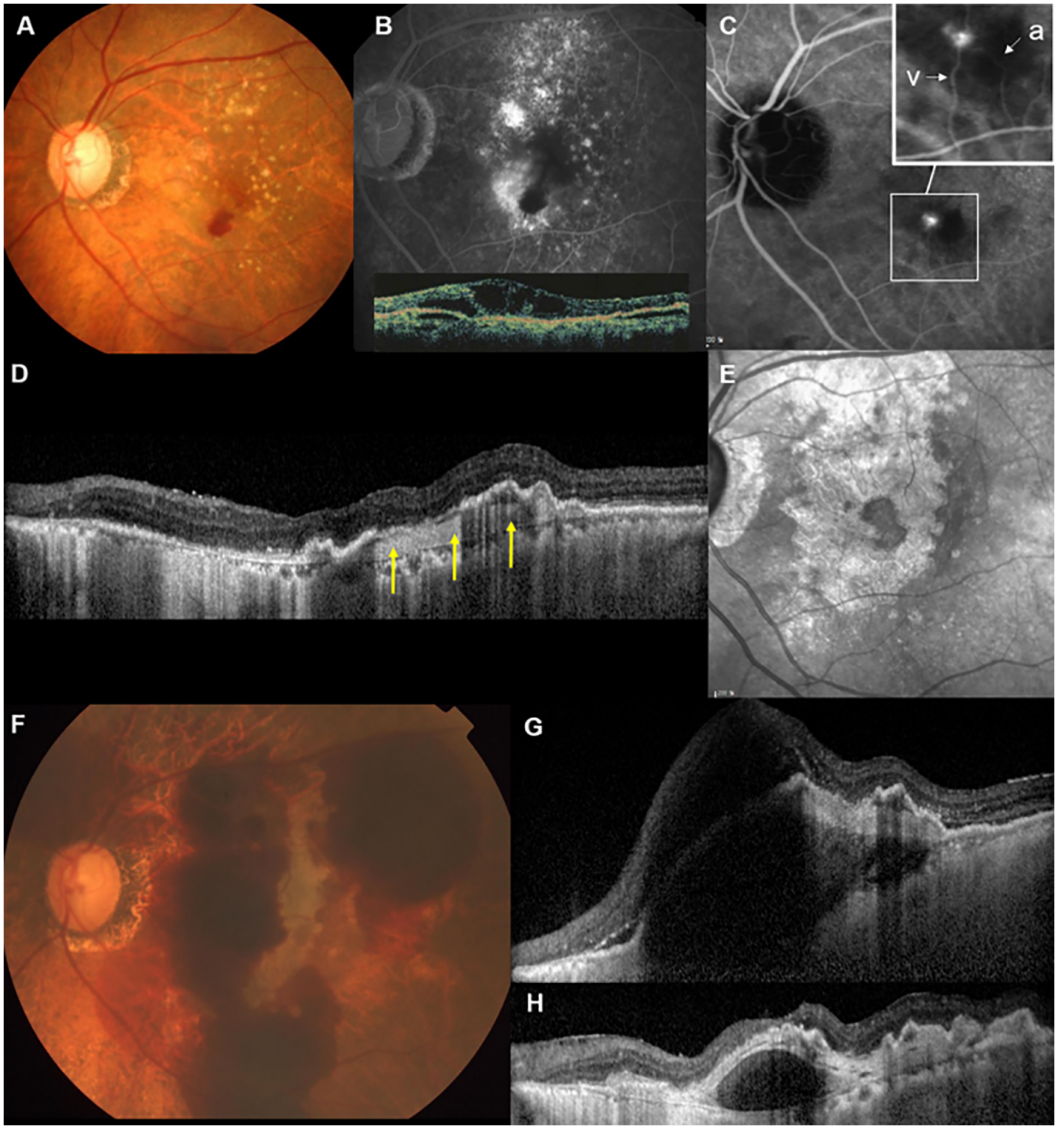
Massive subretinal hemorrhage (SRH) in patient 14, a 69-year-old female with retinal angiomatous proliferation (RAP). (A) At baseline (July 2007), the color fundus photograph showed multiple drusen and retinal hemorrhages inferior to the fovea. (B) Fluorescein angiography (FA) showed intraretinal leakage and blocked fluorescence due to retinal hemorrhage. Inset: Vertical scan through the fovea on Stratus optical coherence tomography (OCT) demonstrated cystic space in the neurosensory retina and pigment epithelium detachment (PED). (C) Mid-to-late phase indocyanine green angiography (ICGA) revealed a small hot-spot lesion, in which an anastomosis between the retinal arteriole (a) and venule (v) was detected on mid-phase ICGA (inset). Best-corrected visual acuity (BCVA) of the left eye was 20/40, and the patient received a combination treatment of photodynamic therapy and bevacizumab injection. After the initial treatment, six ranibizumab and eight bevacizumab injections were performed during the follow-up period; however, the BCVA decreased to 20/200 at 97 months after the initial treatment. (D) Sectional spectral domain (SD)-OCT of the fovea taken at that visit showed well-organized layered lamellar tissue within fibrovascular PED (arrows) as well as atrophic retinal pigment epithelium (RPE) and increased signal transmission in the choroid. (E) Near-infrared images confirmed the development of geographic atrophy during the follow-up period. (F) One month later, massive SRH extending to the major vascular arcade developed. (G) SD-OCT showed subretinal and sub-RPE hemorrhage involving the fovea. The patient received a single bevacizumab injection with gas/rt-PA injection, and 6 months later, BCVA decreased to hand motion. (H) SD-OCT showed the contraction of layered lamellar tissue, and as a result, hyporeflective space was noted, separating the neovascular tissue from the underlying choroid and Bruch’s membrane.

**Fig 3:**
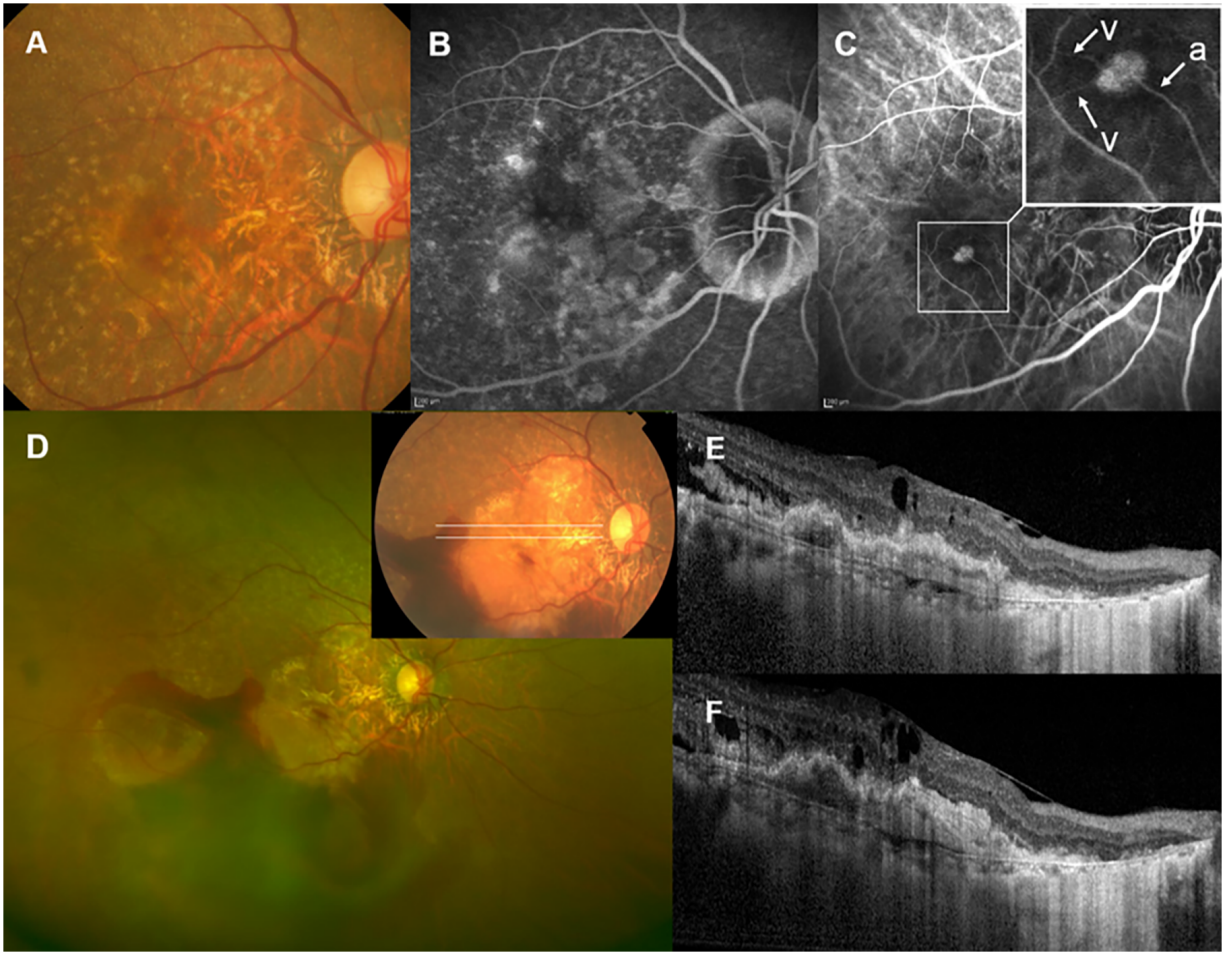
Massive subretinal haemorrhage (SRH) in Patient 11, a 71-year-old woman with retinal angiomatous proliferation (RAP). (A) At baseline, the color fundus photograph showed multiple drusen, retinal hemorrhage, and a neovascular membrane. (B) Fluorescein angiography revealed dye leakage from the neovascular lesion and cystoid changes at the fovea. (C) Mid-phase indocyanine green angiography with magnification (inset). A neovascular complex with retinochoroidal anastomosis composed of a perfusing retinal arteriole (a) and two draining venules (v) was noted. (D) Massive SRH extending past the equator occurred at 21 months after the baseline, and 11 months after the last ranibizumab injection. Best-corrected visual acuity of the right eye decreased from 20/100 to 20/1000. (E) Spectral-domain optical coherence tomography images corresponding to the white lines on color fundus photography (inset of D) revealed intraretinal edema with layered lamellar tissue within fibrovascular tissue under the retinal pigment epithelium.

OCT at the last visit prior to the massive SRH showed widespread fibrovascular tissue under the RPE in 17 of 18 eyes, without any intraretinal/subretinal fluid in 16 eyes. Spectralis OCT was performed at this visit in 13 eyes, and interruption of the RPE line (atrophic RPE layer) was noted within an area of widespread fibrovascular tissue in all 13 eyes. A well-organized layered lamellar band was seen within fibrovascular tissue in 9 of 13 eyes. Representative cases are shown in Figs 2 and 3.

**Fig 4:**
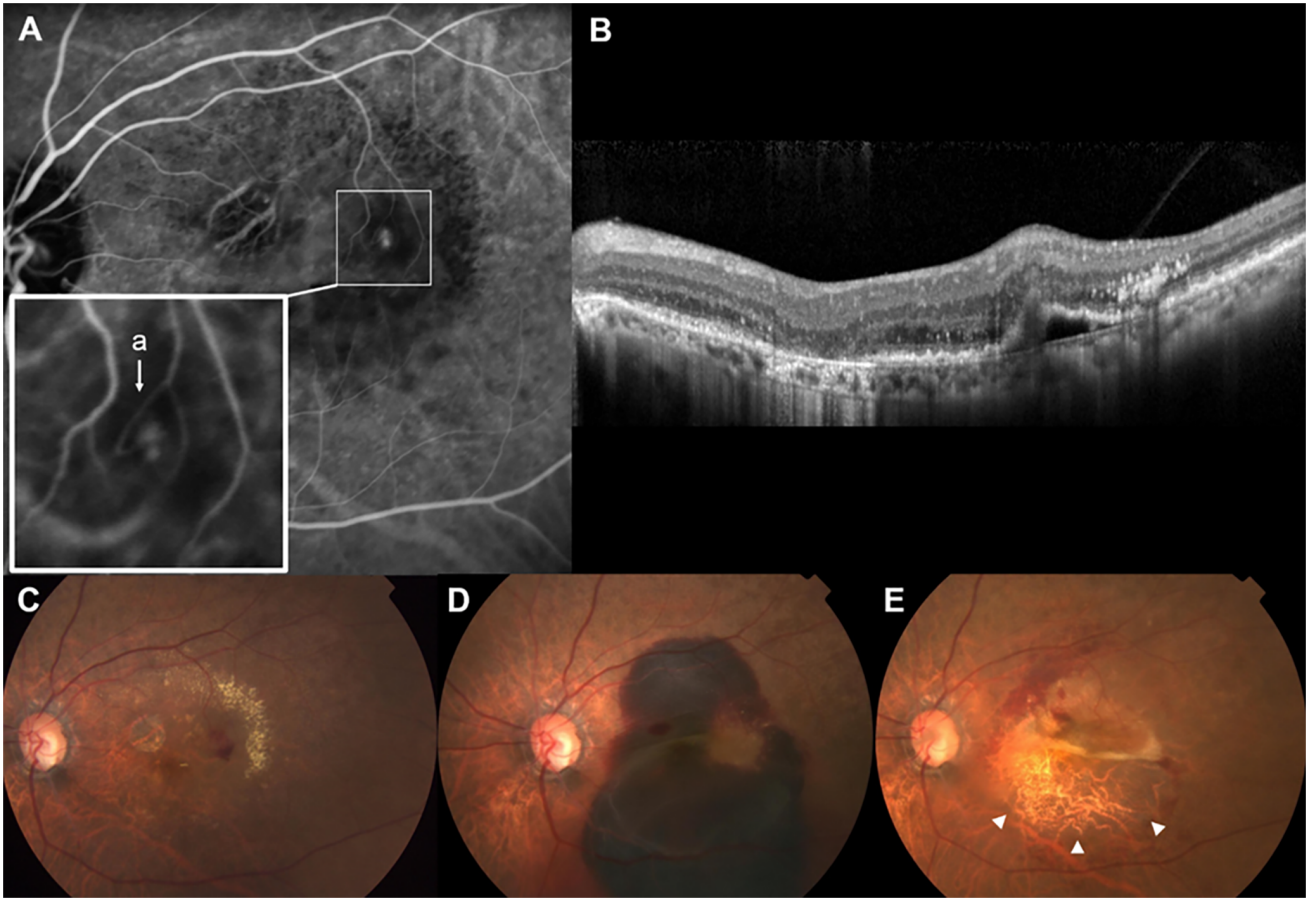
Development of a retinal pigment epithelium (RPE) tear after massive subretinal hemorrhage (SRH) in Patient 3, a 76-year-old woman with retinal angiomatous proliferation (RAP). (A) Mid-to-late phase indocyanine green angiography (ICGA) at baseline revealed a small hot-spot lesion. Inset: Early phase ICGA showing a vascular anastomosis involving retinal arteriole (a). (B) Spectral-domain optical coherence tomography through the lesion demonstrated intraretinal hyperreflective lesion with pigment epithelium detachment. (C) Color fundus photograph at baseline showing retinal hemorrhage and exudate deposition around the hot-spot lesion on ICGA. (D) Massive SRH developed 12 months after the baseline. The patient received a single bevacizumab injection with gas/rt-PA injection. (E) Then, 2 months later, an RPE tear developed (arrowheads) and best-corrected visual acuity of the left eye decreased to counting fingers.

In total, 14 eyes received intravitreal anti-VEGF with gas/rt-PA injections, and four eyes were treated with anti-VEGF injections only. Treatment decision was made at the discretion of a single physician (WK Lee) considering the height of SRH under the fovea, visual acuity at the last visit prior to the massive SRH, and whether the patient can maintain a prone position. In 10 (55.6%) eyes, massive SRH resulted in vitreous hemorrhage, and these eyes underwent pars plana vitrectomy. During the follow-up period, RPE tears (a bare choroid with elevation or scrolling of the torn RPE flap on fundus photography and OCT), which were not seen initially with massive SRH, were noted in six eyes after the absorption of massive SRH (Fig 4).

The median BCVA was 20/100 (range: 20/200–20/32) at baseline, decreasing to 20/200 (range: 20/1000–20/40) at the last visit prior to the massive SRH. All except one patient (case 10) had vision worse than 20/1000 at the final visit after the massive SRH. The clinical characteristics of the patients and the treatment results are summarized in the Table 2.

**Table 2:** Clinical characteristics of 18 eyes with massive subretinal hemorrhage in retinal angiomatous proliferation.

Case No.	Sex/Age at onset	Eye	HTN	F/U (months)	Interval to SRH (months)			No. of anti-VEGF injection prior to SRH	Involvement of retinal arteriole	layered lamellar tissue on SD-OCT prior to SRH	BCVA			Pneumatic displacement	ppV	RPE tear	Others
					From baseline	From the last anti-VEGF injection	From the last stable examination				Base-line	At the visit prior to SRH	Last follow up				
1	M/66	L	Y	24	9	6	2	8	Y	NA	20/50	20/63	LP	Y	N	N	aspirin
2	F/73	R	Y	41	13	2	1	10	Y	NA	20/200	20/100	20/1000	Y	Y	N	aspirin
3	F/76	L	N	55	12	3	2	5	Y	NA	20/32	20/50	CF	Y	N	Y	
4	F/65	L	N	78	37	3weeks	NA	9	Y	NA	20/100	20/160	HM	Y	Y	N	
5	M/65	L	N	90	50	9	1	15	undetectable	Y	20/100	20/500	LP	Y	Y	N	initial PDT
6	F/76	L	Y	50	8	5	3	5	Y	Y	20/160	20/320	LP	N	N	N	aspirin
7	F/71	L	N	78	51	9	3	7	Y	Y	20/40	20/200	HM	N	Y	Y	
8	M/84	R	N	66	6	3days	NA	3	Y	NA	20/63	20/50	LP	Y	Y	Y	
9	M/85	L	Y	60	15	2	1	9	undetectable	N	20/32	20/40	HM	Y	Y	N	aspirin
10	F/70	L	Y	37	6	3	2	5	Y	N	20/160	20/200	20/100	Y	N	N	aspirin
11	F/71	R	N	45	21	11	3	11	Y	Y	20/100	20/1000	HM	Y	Y	N	
12	F/74	L	N	69	48	22	2	6	Y	Y	20/200	20/1000	HM	N	Y	Y	
13	F/71	R	N	82	20	3	2	9	undetectable	N	20/200	20/500	CF	N	N	N	
14	F/69	L	Y	104	98	5	1	14	N	Y	20/40	20/200	HM	Y	Y	N	initial PDT
15	F/79	L	N	50	41	18	1	4	Y	Y	20/63	20/500	20/1000	Y	N	Y	
16	F/81	R	Y	54	51	20	3	8	Y	N	20/100	20/1000	CF	Y	N	N	aspirin
17	F/68	L	Y	59	14	4	1	8	undetectable	Y	20/200	20/250	CF	Y	N	Y	
18	F/72	R	N	81	48	4	1	17	Y	Y	20/32	20/80	LP	Y	Y	N	

HTN = hypertension; F/U = follow-up; SRH = subretinal hemorrhage; VEGF = vascular endothelial growth factor; SD OCT = spectral domain optical coherence tomography; BCVA = best-corrected visual acuity; ppV = pars plana virectomy; RPE = retinal pigment epithelium; LP = light perception; HM = hand movement; CF = counting fingers; PDT = photodynamic therapy

Among 187 eyes of 135 patients, 169 (92.0%), 4 (2.1%), and 11 (5.9%) eyes were analyzed with Spectralis, Cirrus, and Stratus OCT, respectively, until their final visit. Cox regression analysis of 169 eyes analyzed with Spectralis OCT revealed a statistically significant association of massive SRH with the layered lamellar tissue complex on OCT (hazard ratio, HR = 5.883; 95% confidence interval, CI = 1.524–22.713; P = 0.010). No other variable, such as age, hypertension, baseline BCVA, use of PDT, reticular pseudodrusen, or fibrovascular tissue under the RPE at final visit, showed a significant association with the occurrence of massive SRH (Table 3).

**Table 3:** Cox regression analysis of the risk factors for massive submacular hemorrhage in retinal angiomatous proliferation.

Risk factors	Hazard ratio (95% CI)	P value
Age of onset (years)	0.945 (0.857–1.041)	.253
Hypertension (n, %)	1.545 (0.466–5.127)	.477
Initial BCVA (logMAR)	0.715 (0.235–2.172)	.554
Reticular pseudodrusen (n, %)	0.879 (0.248–3.111)	.842
Use of PDT (n, %)	0.199 (0.029–1.367)	.199
Fibrovascular tissue on SD OCT at final visit	4.011 (0.396–40.657)	.240
Well-organized layered lamellar tissue within fibrovascular tissue on SD OCT at final visit	5.883 (1.524–22.713)	.010

BCVA = best-corrected visual acuity; logMAR = logarithm of the minimal angle of resolution; PDT = photodynamic therapy; SD OCT = spectral-domain OCT

## Discussion

**Fig 5:**
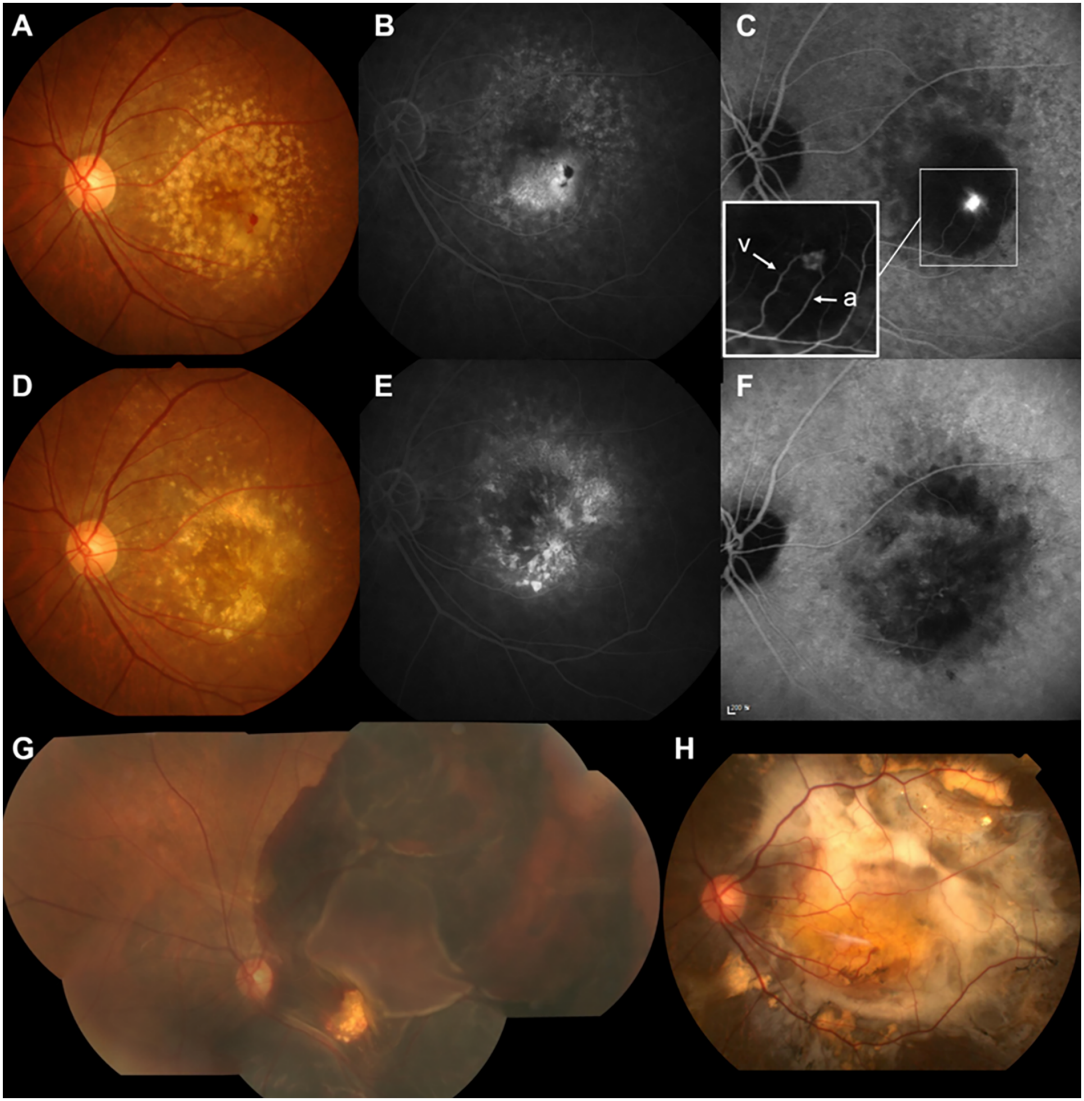
Enlargement of widespread fibrovascular tissue (disease progression) before the development of massive subretinal hemorrhage (SRH) in Patient 4, a 65-year-old woman with retinal angiomatous proliferation (RAP). (A,B,C) Color fundus photograph, fluorescein angiography (FA) and indocyanine green angiography (ICGA) at baseline (Sept. 2006). (C) Late-phase ICGA revealed a hot spot lesion with intraretinal leakage and pigment epithelial detachment. Inset: Early phase ICGA showing a vascular anastomosis between the retinal arteriole (a) and venule (v). (D,E,F) Color fundus photograph, FA and ICGA taken 33 months after baseline. Eight ranibizumab injections were performed during the follow-up period, and best-corrected visual acuity (BCVA) of the left eye decreased, from 20/100 to 20/160. (F) Late-phase ICGA revealed a large plaque lesion at the macula, indicating the enlarged fibrovascular tissue complex. (G) The patient received another ranibizumab injection 3 months later, and massive bullous SRH extending past the equator developed 3 weeks after the last injection. Then, 1 month later, breakthrough vitreous hemorrhage occurred and a pars plana vitrectomy was performed. (H) At the final visit (78 months after baseline, Mar. 2013), BCVA of the left eye was hand motion due to a diffuse submacular fibrous scar.

This is the first study to report the incidence of massive SRH in eyes with RAP lesions (9.6%). The incidence increased steadily, from 2.8% at year 1 to >10% after the 5-year period of treatment and follow-up. In the 18 eyes of our study, massive SRH occurred at the advanced stages of the disease. All except one eye showed widespread fibrovascular tissue under the RPE at the visit prior to the massive SRH, indicating disease progression despite treatment primarily with anti-VEGF drugs (Fig 5). The median BCVA also worsened from baseline until the occurrence of the massive SRH. No eye presented with massive SRH at initial presentation, and the median interval between baseline and the massive SRH was approximately 2 years. This observation somewhat differs from the clinical characteristics of SRH in PCV, which is often found at the initial presentation.

The proposed mechanism of SRH in PCV involves the rupture of polyps, which may occur before any treatment by an increased tension within the polyps during the natural exudative course of the disease.[23] In contrast, massive SRH occurred in RAP eyes that were being treated with chronic and repeated anti-VEGF treatments. Nine of the 13 eyes that underwent Spectralis OCT before the massive SRH showed well-organized layered lamellar bands within the fibrovascular tissue under the RPE, which showed a significant association with the occurrence of massive SRH in a Cox regression analysis. This structure was termed “multilayered PED” by Rahimy and associates.[24] They assumed that this hyperreflective band develops within a chronic fibrovascular PED after repeated anti-VEGF suppression, and consisted of fibrocellular tissue. This fibrous tissue may exert contractile force on the fragile neovascular vessels, which could lead to rupture of these vessels, resulting in massive SRH. RPE tears observed after absorption of the hemorrhage in about one-third of our patients support this. The mechanism of RPE tearing is postulated to involve rapid involution and contraction of neovascular tissue adherent to the undersurface of the RPE.[25]

It is noteworthy that retinal arterioles were involved in the RAP lesion in 13 of 14 (92.8%) eyes with discernable vascular anastomosis on ICGA. A limited number of studies have investigated the nature of the vascular anastomosis found in RAP.[26–28] The involvement of retinal veins ranged from 60 to 82.1%, while the involvement of retinal arteries ranged from 17.6 to 40%, which was 47.5% in eyes without massive SRH in our data. The percentage of arteriole involvement of vascular anastomosis in eyes with massive SRH was notably higher than in eyes without massive SRH and previous studies. The hydrostatic pressure of retinal arteries entering the eye is approximately 60–70 mmHg, and the pressure of the retinal vein is equal to the intraocular pressure (IOP). The hydrostatic pressure of choriocapillaris is 7–8 mmHg higher than the IOP [29], and the pressure gradient between the retinal and choroidal vessels in retinal-choroidal anastomosis may be higher when retinal arterioles are involved in the anastomosis, compared with the involvement of retinal venules. It is therefore possible that the large pressure gradient between the retinal arteriole and neovascular lesion may be one of the factors contributing to the development of massive SRH in RAP.

The use of intravitreal injections of anti-VEGF agents has resulted in a significant improvement of visual outcomes for patients with RAP; however, the optimal dosing regimen still remains to be determined. The most widely used dosing strategy in clinical practice involves a variety of as-needed regimens and treat-and-extend regimens.[30,31] An advantage of the treat-and-extend regimen compared with the as-needed regimen is that it can reduce the risk of SRH during the injection-free interval because of a mandated injection at each visit.[32] In contrast, additional injections with this regimen might increase the risk of contraction of fibrovascular tissue and development of hemorrhage; however, only 2 of 18 eyes developed massive SRH within 1 month after the last anti-VEGF injection in our series. Most of the eyes were not receiving anti-VEGF injections because of apparently stable disease status. The median time between the last injection and the massive SRH was 5 months, and all eyes developed sight-threatening SRH within 3 months of a stable clinical examination and OCT, indicating the need for proactive treatment in these cases. Another concern regarding a proactive regimen is that more frequent injections may accelerate the development of GA. RAP has been identified as a risk factor for GA development following anti-VEGF treatment. However, there is a controversy regarding any association between the number of injections given and the GA growth rate, and at present, there are insufficient data to determine this.[33,34] Because massive SRH in RAP resulted in severe vision loss in most of our cases, it would be beneficial to use the treat-and-extend regimen for the prevention of hemorrhages in RAP eyes with a high risk of a massive SRH.

Our study had several limitations, including its retrospective nature. The initial treatment regimens differed among patients and two patients received PDT initially. However, the intervals between the last PDT and SRH were too long in these patients to suggest a direct effect of PDT on massive SRH. Since we could not collect that data from all enrolled RAP patients, the use of antiplatelet and/or anticoagulation therapy was not included in Cox regression analysis. Although antiplatelet/anticoagulation drugs could be a risk of ocular hemorrhage, the association between such drugs and SRH in nAMD has not been established in large clinical studies yet. [17,35] Finally, involvement of the retinal arteriole was not included in the Cox regression analysis because vascular anastomosis was not discernable in about 30% of all cases. However, the percentage of retinal arteriole involvement in eyes with massive SRH was higher than in previous reports with larger cohorts and that of eyes without massive SRH in our series, so we consider that it may be a contributing factor to the development of massive SRH in RAP.

To our knowledge, this is the first report on the incidence of massive SRH with a large RAP-only cohort, and we collected a relatively large number of cases with massive SRH which was previously considered as an uncommon complication. We performed a survival analysis using follow-up data, which ranged up to 11 years from the initial presentation. The cumulative incidence rate of massive SRH in RAP increased steadily with time and massive SRH usually occurred during injection-free intervals, in the chronic, advanced stages of RAP. We assume that layered lamellar tissue complexes within fibrovascular tissue under the RPE and involvement of retinal arterioles were contributing factors in the development of massive SRH. Because the incidence of massive SRH in RAP is not rare, and the clinical course is devastating even after many different treatments, proactive dosing of an anti-VEGF agent may be a more appropriate approach in RAP eyes at high risk. Additionally, identification of the precise mechanisms, risk factors, and management of massive SRH in RAP should be investigated in future studies.
